# Selective splenic artery embolization for the treatment of thrombocytopenia and hypersplenism in paroxysmal nocturnal hemoglobinuria

**DOI:** 10.1186/1756-8722-7-27

**Published:** 2014-03-27

**Authors:** David J Araten, Anna Paola Iori, Karen Brown, Giovanni Fernando Torelli, Walter Barberi, Fiammetta Natalino, Maria Stefania De Propris, Corrado Girmenia, Filippo Maria Salvatori, Orly Zelig, Robin Foà, Lucio Luzzatto

**Affiliations:** 1 Division of Hematology, NYU School of Medicine, and the New York Harbor VA Medical Center, New York, NY, USA; 2 Department of Hematology, “Sapienza” University of Rome, Rome, Italy; 3 Department of Radiology Memorial Sloan Kettering Cancer Center, New York, NY, USA; 4 Department of Radiological Sciences, Vascular and Interventional Unit, “Sapienza” University of Rome, Rome, Italy; 5 Department of Haematology, Hadassah Medical School, Jerusalem, Israel; 6 Istituto Toscano Tumori, Firenze, Italy; 7 Laura and Isaac Perlmutter Cancer Center, NYU Langone Medical Center, 240 East 38th Street, 19th floor, New York, NY 10016, USA

**Keywords:** PNH, Thrombosis, Selective Splenic Artery embolization, Hypersplenism

## Abstract

**Background:**

PNH is associated with abdominal vein thrombosis, which can cause splenomegaly and hypersplenism. The combination of thrombosis, splenomegaly, and thrombocytopenia (TST) is challenging because anticoagulants are indicated but thrombocytopenia may increase the bleeding risk. Splenectomy could alleviate thrombocytopenia and reduce portal pressure, but it can cause post-operative thromboses and opportunistic infections. We therefore sought to determine whether selective splenic artery embolization (SSAE) is a safe and effective alternative to splenectomy for TST in patients with PNH.

**Methods:**

Four patients with PNH and TST received successive rounds of SSAE. By targeting distal vessels for occlusion, we aimed to infarct approximately 1/3 of the spleen with each procedure.

**Results:**

Three of 4 patients had an improvement in their platelet count, and 3 of 3 had major improvement in abdominal pain/discomfort. The one patient whose platelet count did not respond had developed marrow failure, and she did well with an allo-SCT. Post-procedure pain and fever were common and manageable; only one patient developed a loculated pleural effusion requiring drainage. One patient, who had had only a partial response to eculizumab, responded to SSAE not only with an improved platelet count, but also with an increase in hemoglobin level and decreased transfusion requirement.

**Conclusions:**

These data indicate that SSAE can decrease spleen size and reverse hypersplenism, without exposing the patient to the complications of splenectomy. In addition, SSAE probably reduces the uptake of opsonised red cells in patients who have had a limited response to eculizumab, resulting in an improved quality of life for selected patients.

## Background

PNH is characterized by the triad of hemolytic anemia, cytopenias and a high risk of venous thrombosis. This is a result of the expansion of a stem cell clone with an acquired mutation in the *PIG-A* gene [1], resulting in defective synthesis of glycosylphosphatidylinositol (GPI), and therefore a deficiency of all GPI-linked proteins on the surface of circulating blood cells. Since two complement inhibitors, CD55 and CD59, are GPI-linked, this results in intravascular complement-mediated lysis of red cells. A similar defect on the surface of platelets probably contributes to the marked thrombophilic state of these patients, which particularly involves the intra-abdominal veins [2-7].

Splenomegaly, when it occurs in PNH, is commonly a consequence of splenic, hepatic, or portal vein thrombosis; this can result in hypersplenism and can contribute to cytopenias. We have particularly noted the combination of thrombosis, splenomegaly, and thrombocytopenia (which we term TST) in patients with PNH to be challenging, because splenectomy is hazardous in the presence of portal hypertension, and it may be complicated by further thrombosis [8]. Selective splenic artery embolization (SSAE) represents a potentially safer alternative, available since the 1970’s, when Spigos *et al*[9] used this approach to treat renal transplant patients with hypersplenism and persistent leucopenia. Subsequently, SSAE was used in children [10] and in adults with hypersplenism from biliary atresia [11], thalassemia [12,13], and idiopathic thrombocytopenia purpura [14]. Of note, this approach has been favorably compared with splenectomy in a select group of patients in a randomized trial [15]. To our knowledge, only one patient with PNH—who had complications of a liver transplantation for Budd Chiari syndrome—has previously been treated in this way [16], which resulted in a decrease in ascites. Here we describe four patients with PNH, all of whom had splenomegaly, intra-abdominal thromboses and severe thrombocytopenia, who were treated with SSAE. Three of the patients had a substantial and sustained clinical improvement, and the fourth patient was successfully treated with an allogeneic stem cell graft.

## Results

All 4 patients had hemolytic PNH at the time of SSAE treatment. The main findings and hematologic parameters are shown in Table 1. Here we give brief summaries.

**Table 1:** Clinical and laboratory characteristics of the patients before and after SSAE procedures

**Pt**	**Age at PNH diagnosis (year)**	**Spleen size in cm before SSAE**	**Abdominal pain prior to procedure**	**Representative blood counts before SSAE**			**% GPI-negative blood cells**			**SSAE procedures**			**Plt count post treatment**	**Spleen size in cm post procedure**	
				**HGB g/dl**	**Abs Neut × 10**^**-3**^**/μl**	**Plts × 10**^**-3**^**/μl**	**PMNs**	**RBCs**	**Bone marrow findings**	**Yr of first procedure**	**Number of procedures**	**Complications**			**Follow-up, yrs**
1	30 (1993)	21	Frequent attacks	10.5	1.4	42	52	15	Hypercellular; megakaryocyte aggregates	2000	3	Severe left-sided abdominal pain	123	12	12
2	18 (1990)	19	Frequent attacks	10	2.3	19	95	72	Hypocellular; megakaryocytes reduced	2005	2	Pain; large left sided pleural effusion, resolved with thoracentesis,	12	12	7*
3	27 (1989)	36	No	10.3	1.5	14	75	47	Erythroid hyperplasia; megakaryocytes adequate	2001	1	Abdominal pain	35	NA	11
4	26 (1998)	22	Occasional attacks	7.7	0.9	<10	97	55	Normocellular; megakaryocytes adequate	2009	3	Uneventful	80-100	12	3.5

*In 2009 this Patient underwent successful allogeneic stem cell transplantation from an unrelated donor.

*Patient 1* was referred to us in 1999 with a diagnosis of PNH complicated by DVT (Table 1) and a sonogram showed cavernous transformation of the portal vein, multiple collaterals, markedly attenuated right and middle hepatic veins, with normal flow demonstrated only in the left hepatic vein. She was having frequent attacks of abdominal pain, and in 2000 repeat imaging showed that the spleen had increased to 21.2 cm; endoscopy demonstrated non-bleeding gastric varices but no esophageal varices. The patient was given nadolol to reduce portal pressure and therapeutic doses of low molecular weight heparin.

**Figure 1:**
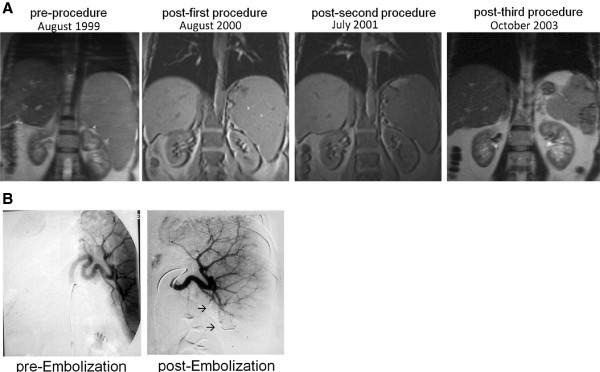
**Patient 1: (A) Serial Coronal MRI’s of the abdomen.** The splenomegaly is seen to progressively resolve, and there is correction of the displacement of the left kidney over time. **(B)** Angiograms of the spleen pre and post procedure, demonstrating insertion of radiopaque material in arterial branches (→) and lack of distal opacification.

The combination of thrombocytopenia, varices, and anticoagulation implied a high long-term risk of hemorrhage, and we considered that the thrombocytopenia was largely the result of hypersplenism. Therefore, in April 2000 the patient underwent selective embolization of the inferior branches of the splenic artery, using gelfoam pledgets and microcoils (Figure 1). The patient developed fever and severe abdominal pain in the LUQ, which subsided within 48 hours. A repeat MR venogram showed a decrease in spleen size to 18.3 cm, with multiple infarcts in the mid to lower pole of the spleen, and the platelet count rose to 61 × 10^3^/ul. A second SSAE procedure was carried out in January 2001, targeting the lower pole of the spleen, as well as some peripheral superior branches. This time there was much less pain; repeat imaging estimated a spleen size of 15 cm, and the platelet count was now in the range from 60 to 80 × 10^3^/ul. Furthermore, there was now almost complete resolution of the abdominal pain. In November 2002 a third SSAE procedure was carried out: this time the 2 remaining major branches of the splenic artery were embolized, as well as a previously embolized branch that seemed to have recanalized. A sonogram estimated the spleen size at 12 cm; the platelet count increased to 162,000 × 10^3^/ul and has been above 100 × 10^3^/ul ever since. The patient is currently on eculizumab and fondaparinux.

**Figure 2:**
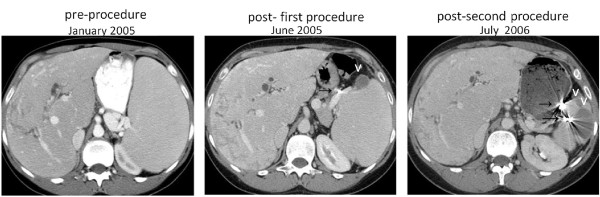
**Patient 2, serial axial CT scans of the abdomen, demonstrating a heterogeneous liver, radiopaque material placed in the branches of the splenic artery (→), which has resulted in wedge shaped splenic infarctions (v).** The splenomegaly is seen to progressively resolve, and there is progressively less impingement upon the left kidney.

*Patient 2* was referred in 1999 because of PNH, an enlarged spleen, and thrombocytopenia (Table 1). Over the previous 4 years she had had 3-6 hospitalizations per year for abdominal pain, and occasional transfusions of packed red blood cells. An MR venogram showed an enlarged liver (18.7 cm) with heterogeneous texture, and thrombosis in the distal right, middle, and left hepatic veins, with attenuation of the proximal hepatic veins, consistent with an early Budd Chiari syndrome. There was also non-occlusive thrombosis in the left portal vein, as well as a narrowed right anterior portal vein. It was considered that thrombosis was too long-standing for thrombolytic therapy [17], and the patient was started on coumadin. By January 2000, the platelet count had fallen to 42 × 10^3^/ul. It was thought that the thrombocytopenia could be due in part to decreased production, and in part to hypersplenism. Therefore, the patient was treated with equine ATG, followed by cyclosporine. Over the next several years there was no improvement in thrombocytopenia, and the patient’s anticoagulation had to be periodically discontinued because of menorrhagia. MR venogram studies demonstrated thrombosis in the left portal vein and splenic vein, as well as splenomegaly. The first SSAE procedure (Figure 2) was complicated by severe pain and a large left sided pleural effusion, which required readmission for thoracentesis and then a VATS procedure for an entrapped lung. Since the platelet count did not change significantly, a second SSAE was carried out, without complications. Following this, the spleen size was reduced, the abdominal pains were greatly alleviated, and the patient was able to return to work full time. However, the thrombocytopenia did not improve and we had to conclude, after a repeat bone marrow, that it had not responded to SSAE because it was due, at least in large part, to decreased platelet production from bone marrow failure. The patient was therefore given rabbit ATG treatment, and did not respond. She then received an unrelated allogeneic SCT, engrafted, and is now in good health.

**Figure 3:**
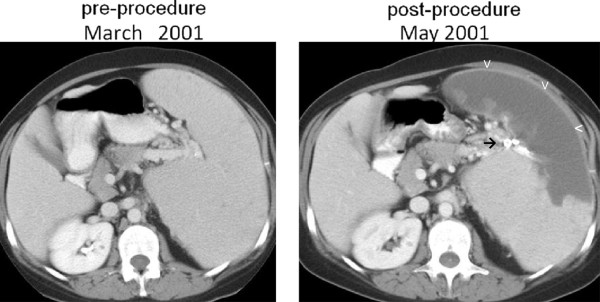
Patient 3, pre and post-procedure axial CT scans with contrast, demonstrating massive splenomegaly, the successful placement of radiopaque material in branches of the splenic artery (→), resulting in splenic infarction (v).

*Patient 3* was referred because of splenic vein thrombosis and an enlarged spleen measuring 36 × 12.5 × 14 cm; a CT showed collateralization of venous flow and some evidence of splenic infarcts. She had very low neutrophil counts, which needed intermittent support with G-CSF. Because the very low platelet counts did not respond well to platelet transfusions, it seemed likely that cytopenias were secondary to splenic sequestration. Therefore, an SSAE procedure was carried out (Figure 3), whereby the lower branch of the splenic artery was embolized by gelatin sponge and stainless steel coils. Approximately ½ of the spleen was thought to have been embolized, and the patient tolerated the procedure well. Within 2 weeks the platelet count had risen to 68,000 × 10^3^/ul, but subsequently it gradually fell to 35,000. Her physicians did not consider that her platelet count was adequate for anticoagulation, and a second embolization was considered but not carried out because she moved overseas. She was then started on eculizumab, which is known to reduce the risk of thrombotic complications of PNH [18], and this also helped her to achieve transfusion-independence. Since the SSAE procedure, the patient has not had any further thrombotic complications. The average platelet count has been 44,000 × 10^3^/ul, whereas the neutrophil count was not affected and has averaged approximately 0.64 × 10^3^/ul.

*Patient 4* was referred in 1998 because of severe hemolytic anemia and a history of superior sagittal sinus thrombosis, and PNH was diagnosed. For 7 years the patient received transfusion support every 5 weeks, and she was maintained throughout her course on oral anticoagulants or low molecular weight heparin. Exacerbations of hemolysis with hemoglobinuria were rare, and overall the patient had a good quality of life. In 2007 the patient reported fatigue, abdominal pain, and headaches. At the same time, the transfusion requirement increased to 1 unit of red blood cells every week. Pancytopenia progressively developed (see Table 1), and no HLA compatible donor was available. Eculizumab was started; fatigue and headaches subsided, but the patient remained transfusion dependent (1 unit of PRC every 3-4 weeks) and she still had abdominal pain. Reticulocytes remained elevated, LDH and unconjugated bilirubin were slightly above normal, and the direct antiglobulin test (DAT) was positive with anti-C3d, whereas the platelet count decreased further to <10 × 10^3^/ul. An abdominal ultrasound study showed marked splenomegaly (22 cm), obliteration of the right and middle hepatic veins and obstruction of the inferior vena cava. It seemed likely that both the thrombocytopenia and the high transfusion requirement were at least to some extent secondary to hypersplenism [19,20] .

**Figure 4:**
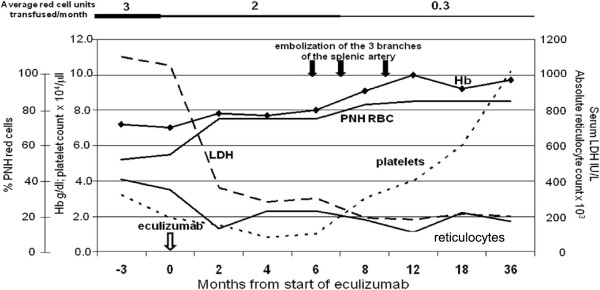
**Time course of hematological parameters of patient 4 in relation to eculizumab treatment and SSAE treatment.** Eculizumab was started in December 2007. SSAE of the three main branches of the splenic artery was performed in June, July, and November 2008. It is seen that the rather severe thrombocytopenia did not respond to eculizumab (nor was that expected), but it did respond to SSAE; the transfusion requirement decreased somewhat on eculizumab but much more after SSAE.

SSAE was performed in three successive procedures carried out in June, July and November 2008, respectively. Each procedure achieved partial embolization of the spleen with coils. Except for a chest infection after the second procedure, the patient tolerated SSAE well. The platelet counts gradually recovered (Figure 4) and three years later stabilized at a level of 80-100,000 × 10^3^/ul. There was also a decrease in reticulocytes, LDH and unconjugated bilirubin. On the other hand, there was a further increase in the percentage of PNH III red cells. These findings, taken together, suggested that extravascular hemolysis was reduced considerably. In keeping with this, the transfusion requirement is now approximately 1 unit of red cells every 3 months; furthermore, her abdominal pains have resolved, and the patient’s quality of life has now improved dramatically.

## Discussion

### SSAE is an effective alternative to splenectomy for TST in PNH patients

All four of our patients had thrombosis, splenomegaly, and thrombocytopenia (TST), a triad that emerges as a syndrome of its own. Splenectomy could be used to treat TST and, by reducing blood flow into the portal system, it could reduce portal pressures. However, splenectomy can be complicated by post-operative thromboses, even in patients who do not have a hypercoagulable state. In addition, total ablation of splenic function is not desirable in patients with PNH, since they may be already neutropenic, may have impaired neutrophil function, and may be immunosuppressed by treatments such as ATG, cyclosporine or eculizumab. SSAE emerges, then, as an alternative to splenectomy, with the aim to reduce spleen function through the occlusion, beyond the hilum, of the branches of the main splenic artery. These branches are end-arteries and therefore this procedure produces infarction of the splenic parenchyma, with consequent reduction of spleen size.

Here we demonstrate that TST is amenable to treatment by SSAE. Following this procedure, all 4 patients had a marked reduction in spleen size, and 3 out of 4 had a clinically significant increase in platelet counts. All 3 of the patients who had had disabling recurrent attacks of abdominal pain or discomfort from splenomegaly reported a substantial improvement in symptoms. We attribute the improvement in the platelet count to a correction of hypersplenism. The mechanism for the relief of pain is less clear: decreased arterial flow to the spleen will decrease the venous return into the portal system, which may ameliorate portal hypertension. Alternatively, decreased spleen size may relieve pressure on the splenic capsule. The clinical and hematological improvement was long-lasting, with a follow-up time of 3.5 to 12 years. The only patient whose thrombocytopenia was not corrected had bone marrow failure in addition to hypersplenism, and therefore she was treated by BMT. Even in her case, the procedure was likely to have been beneficial, because the correction of hypersplenism prior to the BMT may well have facilitated blood count recovery post BMT.

### Radiologic, anatomic, and physiologic considerations pertinent to SSAE

Since the purpose of SSAE is to reduce spleen function, we need to consider the anatomy of the splenic vasculature. The main splenic artery gives off many small branches to the stomach and pancreas before dividing, at the splenic hilum, into several branches. Although there may be a few anastomoses near the hilum, there is little collateral flow within the substance of the spleen: *i.e.*, the branches of the main splenic artery are end-arteries [21]: therefore when one of them is occluded beyond the hilum, the typical result is infarction of the parenchyma that was supplied by that branch.

Our purpose in the use of SSAE in these patients was fundamentally different from that of splenic artery embolization used to arrest hemorrhage secondary to splenic trauma. In that case, the objective is to occlude the vessel proximally in order to diminish the pressure head and facilitate normal hemostasis, while at the same time preserving tissue viability, which is made possible by collateral pathways. In contrast, in our patients, the purpose is to diminish the volume of splenic parenchyma through infarction.

For achieving this, several agents can been used, including gelfoam, polyvinyl alcohol particles, silastic spheres, and balloon catheters. We have preferred gelfoam because it is easy to cut into pledgets small enough to occlude branches of the splenic artery, and because of success reported by several authors. In conjunction with the gelfoam, we also used micro-coils in order to inhibit recanalization. The use of coils for treating hypersplenism by more proximal occlusion of the splenic artery has been abandoned, because the effect on platelet and WBC counts has proven short-lived, on account of the development of collaterals [22].

To avoid infarction of too large a volume of splenic parenchyma (that can result in splenic abscess formation, splenic rupture, pneumonia, and sepsis) we employed a stepwise approach of embolization, aimed to infarct about one-third of the entire spleen, fully aware that we would need to carry out additional rounds. It is preferable to target the lower pole vessels first, in order to limit splinting and thus the development of a left pleural effusion (see Harned *et al.*[23]), which did occur nevertheless in one of our patients.

The most significant clinical side effect of SSAE was severe pain radiating from the left upper quadrant, presumably from nerve endings in the splenic capsule, and it requires medication, including opiates, which, if given in time, can prevent splinting; we recommend delivery intravenously by patient controlled analgesia (PCA). Here, subsequent embolizations were associated with less pain than the initial procedure. Strict aseptic technique is imperative, and we administered peri-procedural antibiotics. Anticoagulants should be stopped prior to the procedure, and then re-started, as we have done, to avoid hemorrhage at the site of arterial entry, and perhaps also to allow a brief period of time for a thrombus to form in the embolized artery. Since splenic rupture is a potential complication, a surgical team with expertise in hepatobiliary diseases should be consulted prior to the procedure and remain available. Because SSAE never removes all of the splenic parenchyma, the risk of overwhelming infection is probably very low; however, pre-procedure vaccinations are reasonable, in the event that complications require a splenectomy. For those on eculizumab, the administration should remain on schedule.

### Role of SSAE in the management of complications of PNH

There are several reasons that the incidence of TST in PNH will decrease over time: (i) eculizumab, a humanized monoclonal antibody against C5, blocks complement-mediated intravascular hemolysis [24] and considerably decreases the risk of thrombosis [18]. In all of our patients, TST developed before eculizumab was available; (ii) primary prophylaxis with anticoagulation – though it is not itself free of risk – can reduce the risk of thrombosis [25]: our patients were referred to us having not been on anticoagulant prophylaxis; (iii) In a group of 9 select patients with recent thromboses, we have shown that thrombolysis is effective for restoring vessel patency [17], and this could prevent TST (none of the 4 patients reported here received thrombolytics on account of their long duration of thromboses); (iv) as eltrombopag is developed as a potential treatment for marrow failure syndromes, it is possible that this will emerge as an alternative treatment for thrombocytopenia in patients with TST. Nevertheless, even in the current era, we expect that there will still be some patients who first present having already had long-standing venous thromboses and TST, and we believe that SSAE will remain an important treatment for them.

Moreover, the clinical course of patient 4 (Figure 4) suggests that here we may have opened up a role for SSAE for a specific subset of PNH patients – those who have had only a partial response to eculizumab. Persistent transfusion dependence occurs in about 15% of patients with hemolytic PNH treated with eculizumab [26]: sometimes this can be explained by rapid drug metabolism, or by marrow failure-- but probably in the majority of cases it is due to extravascular hemolysis primarily in the spleen, as a result of deposition of C3d on red cells, leading to opsonization [19], and very recently, it has been shown that genetic polymorphism of the complement receptor 1 gene is a determinant of response to eculizumab [27]. Indeed, in patient 4, after SSAE, there was not only a marked decrease in spleen size and an increase in platelet counts-- but also a significant reduction in red cell transfusion requirement. We believe that, like the patient reported by Ristano *et al*. who had a good response to splenectomy [20], this patient benefited from a reduction in splenic size—with the advantage that splenic function was not completely lost. If the only indication for SSAE were to decrease splenic uptake of opsonized red cells in those on eculizumab, it will be important to select patients very carefully. One exclusion criterion should be reticulocytopenia (indicative of marrow failure); whereas ongoing transfusion requirement, progressive iron overload, an elevated reticulocyte count and bilirubin level, as well as documentation of C3d on the surface of red cells (based on a Coombs test or flow cytometry) might be regarded as essential inclusion criteria for SSAE in the absence of TST.

## Conclusions

We have shown that SSAE, carried out in sequential steps, is of benefit to patients with PNH who have TST and may have hypersplenism. We have observed clinically significant resolution of pain in all cases, and we have documented objective responses in terms of spleen size and platelet counts in most of the cases. In addition, for patients who remain transfusion dependent on eculizumab on account of extravascular hemolysis, SSAE may provide the extra benefit of alleviating transfusion requirement and is probably preferable to splenectomy.

## Methods

Institutional Review Board approval was obtained for the maintenance of the database from which this paper was written, according to HIPPA guidelines. We identified patients with PNH complicated by TST who were thought to be not amenable to thrombolysis on the basis of long duration of thromboses. Patients who were thought to have a major component of immune mediated marrow failure were either excluded or first treated with immunosuppression prior to the procedure.

A stepwise and selective approach was chosen for embolization. Particularly, we aimed to occlude distal inferior branches of the splenic artery with the first procedure, in order to reduce the chance of a left sided pleural effusion from developing. Our objective was to infarct no more than 1/3 of the spleen with the first procedure and ultimately no more than 2/3 of the spleen overall by the end of the final procedure. Prior to the procedure, anticoagulants were briefly discontinued, peri-procedural antibiotics were administered, and the patients were treated with intravenous fluids until their oral intake recovered. Patients were hospitalized for the procedure, and intravenous opiates-- including delivery by patient controlled analgesia-- was given promptly to relieve pain and prevent splinting, and acetaminophen was given for post-procedure fever.

## Abbreviations

TST: Thrombosis, splenomegaly, and thrombocytopenia; SSAE: Selective splenic artery embolization; PNH: Paroxysmal nocturnal hemoglobinuria; LUQ: Left upper quadrant; DAT: Direct anti-globulin test.

## Competing interests

DJA has served on a scientific advisory board and speakers bureau for Alexion, Inc. LL has served on a scientific advisory board for Alexion in the past and has given unpaid lectures in some Alexion-sponsored events. The other authors have no relevant disclosures.

## Authors’ contributions

DJA, API, and KB contributed equally to this work. DJA, API, KB, RF, and LL conceived the study and wrote the manuscript. These authors, with the assistance of GFT, WB, FN, MSD, CG, and OZ, referred patients to the study and gathered clinical data. KB and FMS performed the radiologic procedures described. All authors read and approved the final manuscript.

## Authors’ information

David J Araten, Anna Paola Iori and Karen Brown: Joint first authors.

## References

[B1] Miyata T Takeda J Iida Y Yamada N Inoue N Takahashi M Maeda K Kitani T Kinoshita T The cloning of PIG-A, a component in the early step of GPI-anchor biosynthesis Science 1993 259 1318 1320 10.1126/science.7680492 7680492

[B2] Araten D Thaler H Luzzatto L High incidence of thrombosis in African-American and Latin-American patients with Paroxysmal Nocturnal Haemoglobinuria Thromb Hemost 2005 93 1 88 91 10.1160/TH04-06-0391 15630496

[B3] Dixon R Rosse W Thorpe A Mechanism of Complement-Mediated Activation of Human Blood Platelets in Vitro J Clin Invest 1977 59 360 368 10.1172/JCI108648 833281 PMC333367

[B4] Jin J Tooze J Marsh J Gordon-Smith E Glycosylphosphatidyl-inositol (GPI)-linked protein deficiency on the platelets of patients with aplastic anaemia and paoxysmal nocturnal haemoglobinuria: two distinct patterns correlating with expression on neutrophils Br J Haematol 1997 96 493 496 10.1046/j.1365-2141.1997.d01-2047.x 9054654

[B5] Sims P Faioni E Wiedmer T Shattil S Complement Proteins C5b-9 Cause Release of Membrane Vesicles from the Platelet Surface That Are Enriched in the Membrane Receptor for Coagulation Factor Va and Express Prothrombinase Activity J Biol Chem 1988 263 18205 18212 2848029

[B6] Wiedmer J Hall S Ortel T Kane W Rosse W Sims P Complement-Induced Vesiculation and Exposure of Membrane Prothrombinase Sites in Platelets of Paroxysmal Nocturnal Hemoglobinuira Blood 1993 82 1192 1196 7688991

[B7] Zimmerman T Kolb W Human Platelet-Initiated Formation and Uptake of the C5-9 Complex of Human Complement J Clin Invest 1976 57 203 211 10.1172/JCI108261 812888 PMC436640

[B8] Kinjo N Kawanaka H Akahoshi T Tomikawa M Yamashita N Konishi K Tanoue K Shirabe K Hashizume M Maehara Y Risk factors for portal venous thrombosis after splenectomy in patients with cirrhosis and portal hypertension Br J Surg 2010 97 6 910 916 10.1002/bjs.7002 20474001

[B9] Spigos D Jonasson O Mozes M Capek V Partial splenic embolization in the treatment of hypersplenism Am J Roentgenol 1979 132 777 782 10.2214/ajr.132.5.777 107745

[B10] Kumpe D Rumack C Pretorius D Stoecker T Stellin G Partial splenic embolization in children with hypersplenism Radiology 1985 155 356 362 10.1148/radiology.155.2.3885306 3885306

[B11] Ando H Ito T Nagaya M Partial splenic embolization decreases the serum bilirubin levels in patients with hypersplenism following the Kasai procedure for biliary atresia J Am Coll Surg 1996 182 206 210 8603238

[B12] Politis C Spigos D Georrgiopoulou P Vrettou H Economidou I Germenis A Richardson C Papaevangelou G Partial splenic embolization for hypersplenism of Thalessemia major: five year follow-up Br Med J 1987 294 665 667 10.1136/bmj.294.6573.665 3105679 PMC1245729

[B13] Stanley P Shen T Partial embolization of the spleen in patients with Thalassemia JVIR 1995 6 137 142 10.1016/S1051-0443(95)71079-X 7703580

[B14] Miyazaki M Itoh H Kaiho T Ohtawa S Ambiru S Hayashi S Nakajima N Oh H Asai T Iseki T Partial splenic embolization for treatment of chronic idiopathic thrombocytopenic purpura Am J Roentgenol 1994 163 123 126 10.2214/ajr.163.1.8010197 8010197

[B15] Mozes M Spigos D Pollak R Abejo R Pavel D Tan W Jonasson O Partial splenic embolization, an alternative to splenectomy–results of a prospective, randomized study Surgery 1984 96 694 702 6385316

[B16] Chang C Singal A Ganeshan S Schiano T Lookstein R Emre S Use of Splenic Artery Embolization To Relieve Tense Ascites Following Liver Transplantation in a Patient with Paroxysmal Nocturnal Hemoglobinuria Liver Transpl 2007 13 1532 1537 10.1002/lt.21317 17969202

[B17] Araten D Notaro R Thaler H Kernan N Boulad F Castro-Malaspina H Small T Scaradavou A Magnan H Prockop S Chaffee S Gonsky J Thertulien R Tarquini R Luzzatto L Thrombolytic therapy is effective in paroxysmal nocturnal hemoglobinuria: a series of nine patients and a review of the literature Haematologica 2012 97 3 344 352 10.3324/haematol.2011.049767 22133780 PMC3291587

[B18] Hillmen P Muus P Dührsen U Risitano A Schubert J Luzzatto L Schrezenmeier H Szer J Brodsky R Hill A Socié G Bessler M Rollins S Bell L Rother R Young N Effect of the complement inhibitor eculizumab on thromboembolism in patients with paroxysmal nocturnal hemoglobinuria Blood 2007 110 12 4123 4128 10.1182/blood-2007-06-095646 17702897

[B19] Risitano A Marando L Seneca E Rotoli B Hemoglobin normalization after splenectomy in a paroxysmal nocturnal hemoglobinuria patient treated by eculizumab Blood 2008 112 449 451 10.1182/blood-2008-04-151613 18606894

[B20] Risitano A Notaro R Marando L Serio B Ranaldi D Seneca E Ricci P Alfinito F Camera A Gianfaldoni G Amendola A Boschetti C DiBona E Fratellanza G Barbano F Rodeghiero F Zanella A Iori A Selleri C Luzzatto L Rotoli B Complement fraction 3 binding on erythrocytes as additional mechanism of disease in paroxysmal nocturnal hemoglobinuria patients treated by eculizumab Blood 2009 113 17 4094 4100 10.1182/blood-2008-11-189944 19179465

[B21] Hilleren D Kadir S Embolization of the spleen for the treatment of hypersplenism and in portal hypertension 1991 Philadelphia: B.C Decker 493 497

[B22] Wallace S Gianturco C Anderson J Goldstein H Davis J Bree R Therapeutic vascular occlusion utilizing steel coil technique: clinical applications Am J Roentgenol 1976 127 381 387 10.2214/ajr.127.3.381 183520

[B23] Harned R Thompson H Kumpe D Narkewicz M Sokol RJ Partial Splenic Embolization in Five Children with Hypersplenism: Effects of Reduced-Volume Embolization on Efficacy and Morbidity Radiology 1998 209 803 806 9844678 10.1148/radiology.209.3.9844678

[B24] Hillmen P Young N Schubert J Brodsky R Socié G Muus P Röth A Szer J Elebute M Nakamura R Browne P Risitano A Hill A Schrezenmeier H Fu C Maciejewski J Rollins S Mojcik C Rother RP Luzzatto L The Complement Inhibitor Eculizumab in Paroxysmal Nocturnal Hemoglobinuria N Engl J Med 2006 355 12 1233 1243 10.1056/NEJMoa061648 16990386

[B25] Hall C Richards S Hillmen P Primary prophylaxis with warfarin prevents thrombosis in paroxysmal nocturnal hemoglobinuria (PNH) Blood 2003 102 10 3587 3591 10.1182/blood-2003-01-0009 12893760

[B26] Luzzatto L Gianfaldoni G Notaro R Management of Paroxysmal Nocturnal Haemoglobinuria: a personal view Br J Haematol 2011 153 709 720 10.1111/j.1365-2141.2011.08690.x 21517820

[B27] Rondelli T Risitano A de Latour RP Sica M Peruzzi B Ricci P Barcellini W Iori AP Boschetti C Valle V Frémeaux-Bacchi V De Angioletti M Socie G Luzzatto L Notaro R Polymorphism of the complement receptor 1 gene correlates with hematological response to eculizumab in patients with paroxysmal nocturnal hemoglobinuria Haematologica 2013 Epub ahead of print (Sept 13) 10.3324/haematol.2013.090001 PMC3912955 24038027

